# Pathway ch-1 study: sphenopalatine ganglion (spg) stimulation for acute treatment of chronic cluster headache (CCH)

**DOI:** 10.1186/1129-2377-14-S1-P57

**Published:** 2013-02-21

**Authors:** J Schoenen, A May, R Jensen, M Láinez, M Lantéri-Minet, C Gaul, A Goodman, A Caparso

**Affiliations:** 1 CHR de la Citadelle, Liège University, Belgium; 2 Universitäts-Krankenhaus Eppendorf, Germany; 3 Glostrup Hospital, University of Copenhagen, Denmark; 4 Hospital Clinico Universitario, Universidad de Valencia, Spain; 5 Hôpital Pasteur, France; 6 University Duisburg-Essen, Germany; 7 Autonomic Technologies, Inc., USA

## Introduction

The pain and autonomic symptoms of cluster headache result from activation of the trigeminal parasympathetic reflex, mediated through the SPG [1,2]. We aimed to investigate the safety and efficacy of SPG stimulation for the acute treatment of CCH.

## Methods

A multi-center, dose range finding, multiple headache attack (HA), acute treatment study with random insertion of placebo was initiated. All subjects met the ICHD-2 criteria for CCH with a minimum of 4 HA/week. Subjects were implanted with a miniaturized neurostimulator which, along with a controller, provides on-demand SPG stimulation. During the blinded experimental period (EXP), each HA was randomly treated with 1 of 3 therapies: full, sub-perception or placebo stimulation. Pain relief at15 minutes (decrease from ‘moderate’ or ‘severe’ to ‘none’ or ‘mild’ on the 5-point scale) and HA frequency reduction were analyzed.

## Results

Thirty-two subjects were enrolled, 27 completed the EXP. One subject remains in EXP, 1 skipped EXP, 2 were explanted due to early lead migration, and 1 did not complete the implant due to difficult anatomy. Pain relief was achieved in 67% of HA (n=190) treated with full compared to 8% (n=183) with sub-perception and 8% (n=189) with placebo stimulation. A clinically significant improvement occurred in 19 of 27 (70%) subjects: 7 (26%) achieved acute pain relief in ≥50% of treated HAs, 10 (37%) a ≥50% reduction in HA frequency compared to baseline and 2 (7%) experienced both. Of the 12 frequency responders, HA frequency was reduced to ≤2 HA/week in 9 subjects. Eight (29%) of the 27 did not respond or did not provide sufficient data for evaluation. Most subjects (47%) experienced transient, mild to moderate numbness within the second division of the trigeminal nerve post implant with 62% resolving within the first three months.

## Conclusions

Results suggest that acute, on-demand SPG stimulation using the ATI Neurostimulation System has acute and preventive effects and is an effective novel therapy for CCH. In this study, 70% of subjects responded to the therapy.

## References

[B1] Goadsby PJ Pathophysiology of cluster headache: a trigeminal autonomic cephalgia Lancet Neurol 2002 14 4 251 7 Epub 2003/07/10 10.1016/S1474-4422(02)00104-7 12849458

[B2] Goadsby PJ Edvinsson L Human in vivo evidence for trigeminovascular activation in cluster headache. Neuropeptide changes and effects of acute attacks therapies Brain 1994 14 Pt 3 427 34 Epub 1994/06/01 7518321 10.1093/brain/117.3.427

